# Editorial: Regulation of the host’s immune system by parasitic infections and its implications

**DOI:** 10.3389/fcimb.2022.1118692

**Published:** 2022-12-21

**Authors:** Martha Legorreta-Herrera, Miriam Rodriguez-Sosa, Abhay R. Satoskar

**Affiliations:** ^1^ Laboratorio de Inmunología Molecular, Unidad de Investigación Química Computacional, Síntesis y Farmacología de Moléculas de Interés Biológico, Facultad de Estudios Superiores Zaragoza, Universidad Nacional Autónoma de México, Ciudad de México, Mexico; ^2^ Laboratorio de Inmunidad Innata. Unidad de Investigación en Biomedicina, Facultad de Estudios Superiores Iztacala, UNAM, Ciudad de México, Mexico; ^3^ Department of Pathology, Wexner Medical Center, The Ohio State University, Columbus, OH, United States; ^4^ Department of Microbiology, Wexner Medical Center, The Ohio State University, Columbus, OH, United States

**Keywords:** *Plasmodium*, *Schistosoma*, *Leishmania*, *Trichinella*, host-parasite interaction

Protozoa and helminth infections are major health problems worldwide. These infections may become chronic and impair quality of life or cause significant morbidity and mortality. Unfortunately, at present, vaccines have not been developed for every parasitic disease; however, during the last decade, we have progressed our understanding of the development of innate and adaptive immune responses that control or promote the development of parasitic diseases. Initial parasitic infection induces an intense innate inflammatory immune response that regulates parasitic proliferation but also damages the host and could be more harmful than the parasite. The success of the immune response against parasites depends on the fine regulation of the innate inflammatory immune response to efficiently eliminate the parasites without damaging the host, inducing specific protective humoral immunity. Therefore, understanding the mechanisms involved in the host-parasite interactions that affect different cells, signaling molecules, cytokines, hormones, or metabolites is essential for developing efficient therapeutic strategies and vaccines against parasites.

In this Research Topic, several original high-quality research contributions cover different aspects of immune responses and their regulation in some of the most important parasitic diseases in the world. Here, we learn about the influence of testosterone on sexual dimorphism in the immune response to malaria, the role of the cytokine MIF on the innate immune response during *Plasmodium* infection, the identification of potential cytokines and chemokines to differentiate between sepsis and malaria, the participation of the key regulator of immunological processes in the infection of *Leishmania donovani*, the regulation of genes depending on the parasite sex of *Schistosoma mansoni*, and the modulating role of secretion factors by the parasite *Trichinella spiralis* in the regulation of neutrophil function.

Here, Martha Legorreta-Herrera’s group addresses sexual dimorphism in malaria. This original research used *Plasmodium berghei* ANKA infection to demonstrate that testosterone concentration affects the immune response cells and cytokine levels required to eliminate *Plasmodium berghei* ANKA differently for each sex. In females, testosterone administration decreases parasitemia, which prevents weight loss associated with increased CD8^+^ and decreased B220^+^ cell populations. By contrast, males exhibit increased parasitemia and NK cells and decreased IFN-γ levels. These findings partially explain the sexual dimorphism in malaria and suggest possible implications for the efficiency of antimalarial treatments and vaccines (https://pubmed.ncbi.nlm.nih.gov/36237427).

Another original contribution came from the group led by Miriam Rodríguez-Sosa, who demonstrated the critical role played by the cytokine MIF as a regulator of the inflammatory response associated with the pathogenesis and lethality of malaria. MIF knockout mice infected with *Plasmodium yoelii* 17XL showed reduced parasitemia and significantly increased survival compared with infected wild-type mice. The absence of MIF induced an immune response with a mixed Th1/Th2 serum cytokine profile (IL-12, IL-17/IL-4, IL-10, and MIF^-/-^), and macrophages exhibited high levels of MIF, IL-10, and IL-12 and low levels of TNF-α and nitric oxide compared with wild-type macrophages. Thus, they demonstrated that the MIF cytokine is a regulator of the inflammatory immune response associated with the pathogenesis and lethality of malaria (https://pubmed.ncbi.nlm.nih.gov/36093199/).

In sub-Saharan Africa, where malaria is endemic, sepsis also frequently occurs in children; both diseases share clinical features and a dysregulated proinflammatory immune response, complicating differential diagnosis in the absence of confirmatory tests. In this collection, the group of Michael Fokuo Ofori demonstrated that the proinflammatory response is more intense in malaria than in sepsis. They suggest that the cytokines and chemokines IL-1β, IL-7, IL-12, IL-1RA, RANTES/CCL5, MIP1B/CCL4, and IP10/CXCL10 could be used as potential markers to discriminate children with sepsis from those with clinical malaria and other febrile conditions (Frimpong et al.).

A further original contribution came from the group of Shailja Singh, who reported for the first time that SUMOylation is involved in the infection process *of Leishmania donovani*, a causative agent of visceral leishmaniasis. *L. donovani* infection in host macrophages leads to upregulation of SUMOylation-promoted pathway genes and downregulation of a SUMOylation gene, SENP1, which promotes *L. donovani* infection. Deletion of host SUMOylation pathway genes led to reduced expression levels of host autophagy markers while promoting autophagosome-lysosome fusion. Levels of reactive oxygen species, nitric oxide, and proinflammatory cytokines increased with the knockout of host SUMOylation pathway genes during *L. donovani* infection. Therefore, the SUMOylation pathway modulates protective immune responses, promoting parasite survival (Singhal et al.).

Regarding the interaction of helminths with their host, in schistosomiasis (a parasitic disease affecting more than 230 million people), the immune response against *Schistosoma* eggs trapped in tissues induces an inflammatory and fibrotic reaction. Unfortunately, there are no vaccines to prevent infection or antifibrotic drugs. To identify molecular targets to eliminate *Schistosoma mansoni*, Martina Sombetzki’s group addressed the interaction of male or female schistosomes, or a combination of both, with their host through comparative transcriptomic and cytometric analysis of female mouse spleens. From a total of 22,207 transcripts, 1,293 genes were differentially expressed in the male group, 512 in the female group, and 4,062 in the combined male and female group compared with uninfected controls. In addition, they showed that adult male and female helminths trigger sex-specific immune responses independent of those induced by eggs. This information could help the identification of target genes for *Schistosoma* elimination (Winkelmann et al.).

Helminths infect many mammals, including humans; these parasites have evolved different strategies to evade the immune response. *Trichinella spiralis* avoids the destruction of their newborn larvae by host neutrophils by secreting the serine protease inhibitor (TsSERPs) involved in digestion and inflammation, which allows it to survive longer in its host and cause chronic infection. Poom Adisakwattana’s group investigated the immunomodulatory properties of recombinant TsSERPs (rTsSERP1) on neutrophil function. They found that rTsSERP1 inhibits neutrophil elastase, reduces neutrophil phagocytic activity and extracellular neutrophil trap formation, and decreases the synthesis of chemokines and proinflammatory cytokines in activated neutrophils. These immunomodulatory mechanisms could constitute a therapeutic target for inflammatory diseases (Kobpornchai et al.).

Finally, the editors of this Research Topic believe that this collection of articles will serve to help us better understand the regulatory mechanisms of the immune response around a parasitic infection and its possible implications in the regulation of the course of the pathology. We hope these articles will lead a productive discussion and be helpful to researchers actively involved in immunoparasitology, as well as for those who are starting their research in this area, and that they can provide a point of reference.

## Author contributions

ML-H, MS, and AS are guest associate editors of the Research Topic and wrote and edited the manuscript. All authors contributed to the article and approved the submitted version.

